# Monsoon versus Uplift in Southwestern China–Late Pliocene Climate in Yuanmou Basin, Yunnan

**DOI:** 10.1371/journal.pone.0037760

**Published:** 2012-05-22

**Authors:** Yi-Feng Yao, Angela A. Bruch, Ye-Ming Cheng, Volker Mosbrugger, Yu-Fei Wang, Cheng-Sen Li

**Affiliations:** 1 State Key Laboratory of Systematic and Evolutionary Botany, Institute of Botany, Chinese Academy of Sciences, Beijing, China; 2 Heidelberg Academy of Sciences and Humanities, Research Centre ‘‘The Role of Culture in Early Expansions of Humans” at Senckenberg Research Institute, Frankfurt am Main, Germany; 3 Geological Museum of China, Beijing, China; 4 Senckenberg Research Institute, Frankfurt am Main, Germany; University College London, United Kingdom

## Abstract

Yuanmou Basin of Yunnan, SW China, is a famous locality with hominids, hominoids, mammals and plant fossils. Based on the published megaflora and palynoflora data from Yuanmou Basin, the climate of Late Pliocene is reconstructed using the Coexistence Approach. The results indicate a warm and humid subtropical climate with a mean annual temperature of ca. 16–17°C and a mean annual precipitation of ca. 1500–1600 mm in the Late Pliocene rather than a dry, hot climate today, which may be due to the local tectonic change and gradual intensification of India monsoon. The comparison of Late Pliocene climate in Eryuan, Yangyi, Longling, and Yuanmou Basin of Yunnan Province suggests that the mean annual temperatures generally show a latitudinal gradient and fit well with their geographic position, while the mean annual precipitations seem to be related to the different geometries of the valleys under the same monsoon system.

## Introduction

Yuanmou Basin of Yunnan, SW China is famous for the discoveries of hominids and hominoids. In 1965, two hominin incisors were found by Qian Fang from the fourth member of Yuanmou Formation [1], [2]. Later, the two incisors were described and attributed to *Homo erectus yuanmouensis* [1], [3], and were dated to ∼1.7 Ma using magnetostratigraphy and the sedimentation rate, which are the earliest evidence for hominins in East Asia [4]. From the sedimentary layer bearing the hominin fossils, abundant mammalian fossils (e.g. *Nestoritherium* (*Hesperotherium*) sp., *Cervocerus ultimus*, *Procapreolus stenosis*) and pollen (such as *Pinus*, *Alnus*, Asteraceae, and Poaceae) were recovered [5]–[7]. Based on the faunal and palynological assemblages, it is indicated that Yuanmou *Homo* lived in an area with a diversity of habitats, including open grassland, bushland, forest, marsh, and fresh water [4]. Although the occurrence of *Homo erectus* in subtropical SW China is still debated [8], Yuanmou Basin becomes an important site for studying vegetation and climate history, and the influence of environmental changes on early human evolution in SW China.

The Pliocene epoch (5.3–2.6 Ma, [9]) represents a transition from a relatively warm climate stage into the icehouse of the Pleistocene. Earlier, some palaeobotanical and palynological investigations of Pliocene have been conducted in Yuanmou Basin for qualitative reconstruction of the palaeovegetation and palaeoenvironment [6], [10]–[16]. Most of the studies show a warm and humid subtropical climate during that time. However, no quantitative climate analysis for this region is available yet. By applying the Coexistence Approach [17] to the published Late Pliocene flora (including woods, leaves, and pollen) from Yuanmou Basin, this paper aims to quantitatively evaluate the climate in this basin during the Late Pliocene, a period before the occurrence of early hominins, and to determine the differences in climate between the Late Pliocene and today in this region.

## Materials and Methods

### Geological and Geographic Setting

**Figure 1:**
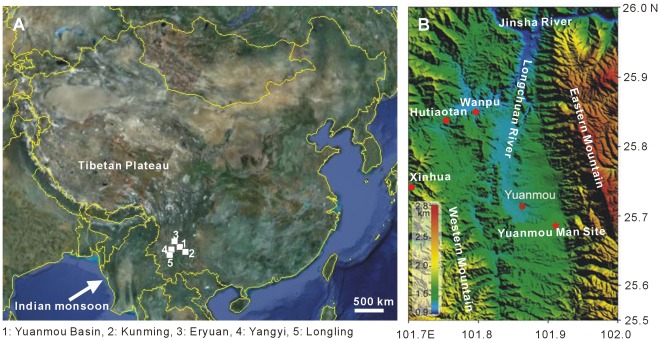
Map showing the position (A, from http://maps.google.com) and topography (B, modified from [**4**]) of Yuanmou Basin, Yunnan, SW China.

Yuanmou Basin is situated at the southeastern margin of the Tibetan Plateau and lies about 110 km northwest of Kunming, Yunnan Province, SW China (Fig. 1A). The basin covers an area of 187 km^2^ with a length of 30 km and a maximum width of 9 km. The elevation of the basin floor ranges from 1000 m to 1400 m above sea level, while the eastern and western mountains reach up to 2200–2800 m and 1200–1800 m, respectively. The Longchuan River flows through the basin from south to north and joins the Jinsha River, the uppermost reaches of the Yangtze River (Fig. 1B).

**Figure 2:**
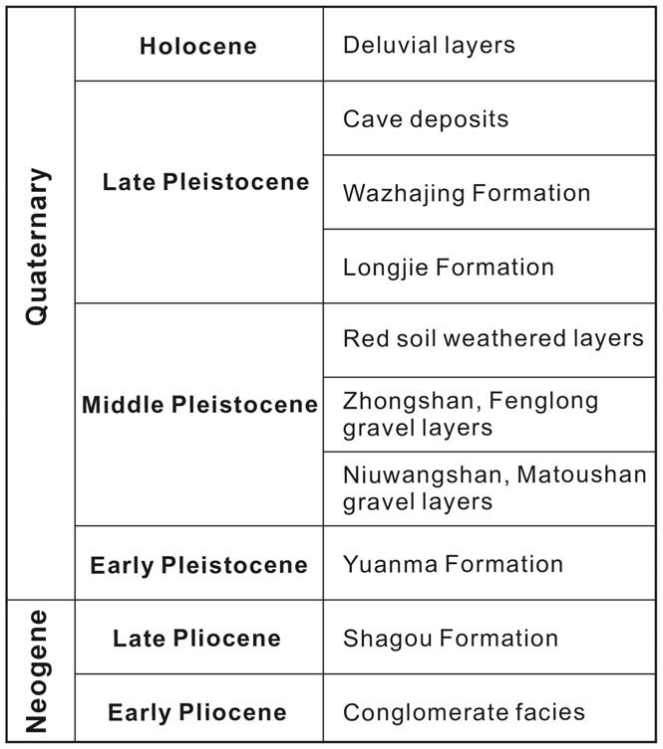
Stratigraphic sequence of Late Cenozoic in Yuanmou Basin.

Yuanmou Basin is a north-south elongated fault basin formed during the uplift process of the Tibetan and Yunnan Plateaus. The basin is bounded in the eastern side by Cretaceous and Jurassic sediments and in the western side by Precambrian sediments [18]. The Late Cenozoic fluvio-lacustrine sediments were well preserved in this basin. Based on the correlation with the lithostratigraphy, biostratigraphy and magnetostratigraphy, the Late Cenozoic strata in Yuanmou Basin are divided into following stratigraphic units in ascending order, namely conglomerate facies of Early Pliocene, Shagou Formation of Late Pliocene, Yuanma Formation of Early Pleistocene, Niuwangshan, Matoushan, Zhongshan and Fenglong gravel layers and red soil weathered layers of Middle Pleistocene, Longjie, Wazhajing Formations and cave deposits of Late Pleistocene, and the deluvial layers of Holocene (Fig. 2) [19]. In the Shagou Formation, the representative mammalian fossils comprise *Stegodon yuanmouensis*, *S. zhaotongensis*, *S. elephantoides*, *S. primitium*, *Stegolophodon banguoensis*, *Serridentinus* sp., *Chilotherium yunnanensis*, *Enhydridon cf. falconeri*, *Rhinoceros* sp., *Cervus* sp., and *Sus* sp. [20].

### Modern Climate and Vegetation

**Figure 3:**
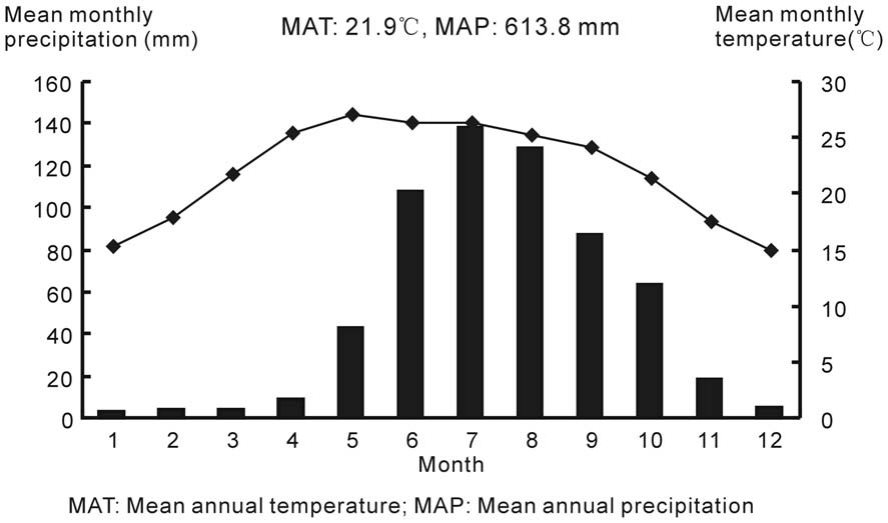
Current climate in Yuanmou.

**Figure 4:**
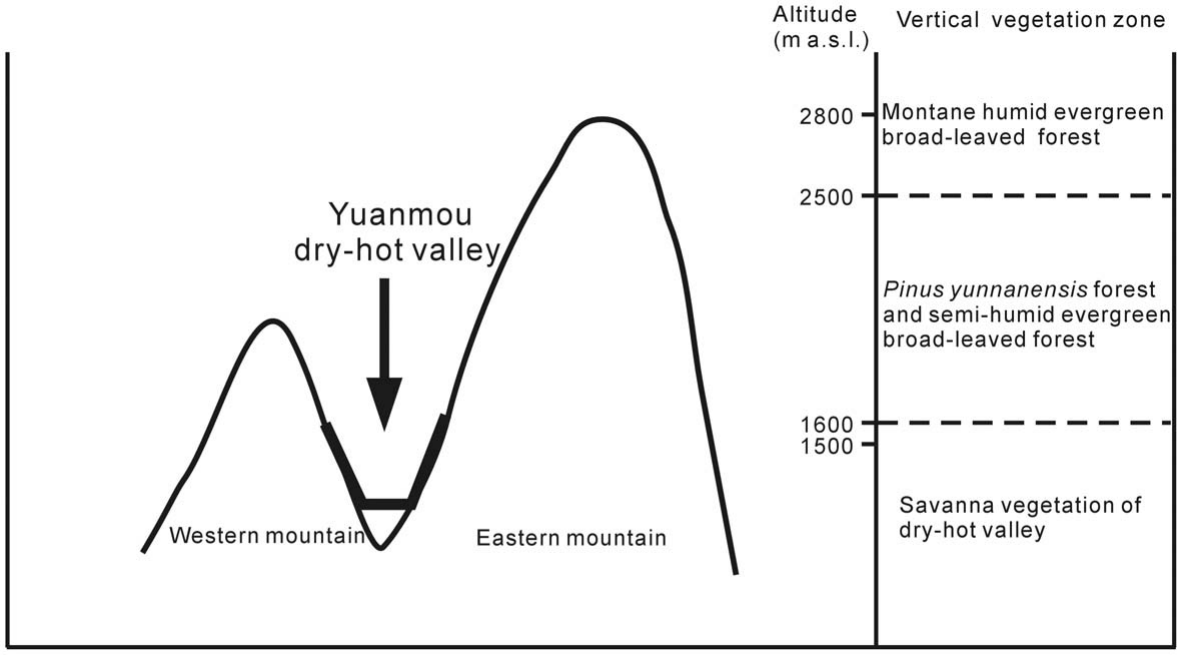
Sketch map showing the relation of modern hypsographic and vegetation of Yuanmou dry-hot valley.

The current climate of this region is a southern subtropical type, which is controlled by the Indian monsoon in summer and by the southern stream of the westerly winds in winter. Mean annual temperature and precipitation are 21.9°C and 613.8 mm, respectively (Fig. 3). More than 80% of the annual precipitation falls in the rainy season (May-October) [21]. The basin is one of the typical dry-hot vallies with a high annual evaporation capacity of ca. 3500 mm, and the foehn effect, viz. Airflow climbs over the mountain and adiabatically sinks at leeward slope causing a temperature rise and humidity reduction, is prevailing in this region. Below 1600 m above sea level, the vegetation is of savanna type of dry-hot valley. Between 1500 m and 2500 m, the vegetation is dominated by semi-humid evergreen broad-leaved forest and *Pinus yunnanensis* forest. Above 2500 m, the vegetation is a type of montane humid evergreen broad-leaved forest (Fig. 4) [22].

### Data Collection and Methodology

For the quantitative climate analysis, fossil woods [13]–[16], leaves [10], [12] and pollen floras [12] from Yuanmou Basin have been compiled from the literature. The fossil woods were collected from the Shagou Formation of Wanpu, Xinhua and Hutiaotan Earth Forest in Yuanmou Basin (Fig. 1B), which belongs to Late Pliocene based on the correlation with the first and second members of Yuanmou Formation dated to 3.4–2.5 Ma by palaeomagnetic dating [7], [13], [19]. The fossil leaves and pollen were recovered from the Gantang Formation of Wanpu, which is assigned to Late Pliocene based on a lithostratigraphic and biostratigraphic comparison with Shagou Formation [12], [18]. All fossil taxa and their Nearest Living Relatives (NLRs) are given in Tables 1 and 2.

**Table 1:** List of megafossils from Yuanmou Basin.

Taxon number	Fossil taxon	NLR	Type of fossil
1	*Ulmus longifolia*	*Ulmus*	leaf
2	*Ulmus* sp.	*Ulmus*	leaf
3	*Ulmus miopumila*	*Ulmus pumila*	leaf
4	*Ulmus yunnanensis*	*Ulmus*	leaf
5	*Ulmus carpinoides*	*Ulmus*	leaf
6	*Ulmus multinervis*	*Ulmus castaneifolia*	leaf
7	*Ulmus hedinii*	*Ulmus*	leaf
8	*Fagus yunnanensis*	*Fagus*	leaf
9	*Typha lesquereuxii*	*Typha*	leaf
10	*Betula mioluminifera*	*Betula*	leaf
11	*Betula angusta*	*Betula*	leaf
12	*Salix masamunei*	*Salix*	leaf
13	*Salix angusta*	*Salix*	leaf
14	*Salix* cf. *varians*	*Salix*	leaf
15	*Cinnamomum* sp.	*Cinnamomum*	leaf
16	*Phoebe* sp.	*Phoebe*	leaf
17	*Myrica yunnanica*	*Myrica*	leaf
18	*Albizia bracteata*	*Albizia bracteata*	leaf
19	*Albizia* sp.	*Albizia*	leaf
20	*Taiwania* sp.	*Taiwania* *cryptomerioide*s	leaf
21	*Litsea grabaui*	*Litsea*	leaf
22	*Alnus protomaxiwiczii*	*Alnus*	leaf
23	*Zelkova ungeri*	*Zelkova*	leaf
24	*Zelkova speciosa*	*Zelkova*	leaf
25	*Corylus* sp.	*Corylus*	leaf
26	*Crataegus yuanmouensis*	*Crataegus*	leaf
27	*Amelanchier wongii*	*Amelanchier sinica*	leaf
28	*Leguminosites climensis*	*Sophora*	leaf
29	*Acer florinii*	*Acer*	leaf
30	*Acer* sp.	*Acer*	leaf
31	*Viburnum ovalifolium*	*Viburnum*	leaf
32	*Graminites* sp.	Poaceae	leaf
33	*Rhododendron* sp.	*Rhododendron*	leaf
34	*Berchemia* sp.	Rhamnaceae	leaf
35	*Podogonium oebningense*	Fabaceae	leaf
36	*Bischofia* cf. *javanica*	*Bischofia javanica*	wood
37	*Cedreloxylon cristalliferum*	*Toona*	wood
38	*Lagerstroemioxylon* *yuanmouensis*	*Lagerstroemia*	wood
39	Taxaceae	*Amentotaxus*	wood
40	Cephalotaxaceae	*Cephalotaxus*	wood
41	*Quercoxylon* sp.	*Cyclobalanopsis*	wood
42	*Zelkovoxylon* sp.	*Zelkova*	wood
43	*Pterocaryoxylon* sp.	*Pterocarya*	wood
44	*Dalbergioxylon* sp.	*Dalbergia*	wood
45	*Albizinium* sp.	*Albizia*	wood
46	*Castanoxylon* sp.	*Castanopsis*	wood

**Table 2:** List of palynomorphs from Yuanmou Basin.

Taxon number	Fossil taxon	NLR
1	*Pinus* sp.	*Pinus*
2	*Tsuga* sp.	*Tsuga*
3	*Keteleeria* sp.	*Keteleeria*
4	*Carpinus* sp.	*Carpinus*
5	*Alnus* sp.	*Alnus*
6	*Betula* sp.	*Betula*
7	Gramineae	Poaceae
8	*Juglans* sp.	*Juglans*
9	*Carya* sp.	*Carya*
10	*Juglans regia*	*Juglans regia*
11	*Tetrocolporites* sp.	Meliaceae
12	*Tricolpites*	Hamamelidaceae
13	*Cyclobalanopsis* sp.	*Cyclobalanopsis*
14	*Quercus* sp.	*Quercus*
15	*Ilex* sp.	*Ilex*
16	*Lithocarpus* sp.	*Lithocarpus*
17	*Castanopsis* sp.	*Castanopsis*
18	*Ulmus* sp.	*Ulmus*
19	*Zelkova* sp.	*Zelkova*
20	*Liquidambar* sp.	*Liquidambar*
21	Ericaceae	Ericaceae
22	*Symplocos* sp.	*Symplocos*
23	*Artemisia* sp.	*Artemisia*
24	*Elaeagnus* sp.	*Elaeagnus*
25	*Caesalpinia* sp.	*Caesalpinia*
26	Caprifoliaceae	Caprifoliaceae
27	Compositae	Asteraceae
28	*Fupingopollenites wackersdorfensis*	Verbenaceae
29	*Polygonum* sp.	*Polygonum*
30	*Scabiosa* sp.	*Scabiosa*
31	*Annamocarya* sp.	*Annamocarya*
32	*Engelhardtia* sp.	*Engelhardtia*
33	*Cyclocarya* sp.	*Cyclocarya*
34	*Inaperturopollenites*	Taxodiaceae, Cupressaceae
35	*Corylus* sp.	*Corylus*
36	*Pterocarya* sp.	*Pterocarya*
37	*Celtis* sp.	*Celtis*
38	*Euphorbia* sp.	*Euphorbia*
39	*Typha* sp.	*Typha*
40	Hamamelidaceae	Hamamelidaceae
41	*Ephedra* sp.	*Ephedra*
42	*Myrica* sp.	*Myrica*
43	*Reveesia* sp.	*Reveesia*
44	*Pittosporum* sp.	*Pittosporum*
45	*Loropetalum* sp.	*Loropetalum*
46	*Altinigia* sp.	*Altinigia*

We consulted the Writing Group of Cenozoic Plants of China [23] and Song et al. [24] in deciding the NLRs. In the present study, the Coexistence Approach has been employed for the quantitative climate analysis of the Late Pliocene floras from Yuanmou Basin. This method can be applied for quantitative terrestrial climate reconstructions in the Cenozoic using plant fossils, including leaves, fruits and seeds, pollen and wood. Based on the assumption that the climatic tolerance of a fossil taxon is similar to that of its NLR, the Coexistence Approach determines the climatic ranges in which a maximum number of NLRs of a given fossil flora can coexist. The coexistence interval is taken as the best estimate of the climatic conditions under which the fossil flora once lived. The detailed procedure for obtaining the climatic tolerance of a NLR follows Yao et al. [25]. Firstly, the climatic parameters of all NLRs in a fossil flora are obtained from the climatic records within their modern distribution area. Secondly, the maximum and minimum of each parameter of each NLR are established. Thirdly, the climatic interval of each parameter of all NLRs is overlapped and the coexistence interval of all NLRs is obtained. Using the Coexistence Approach, the following climatic parameters have been considered for palaeoclimatic analysis, i.e. mean annual temperature (MAT), temperature of the warmest month (WMT), temperature of the coldest month (CMT), mean annual precipitation (MAP), wettest month precipitation (HMP), driest month precipitation (LMP). In addition, the mean annual ranges of temperature are calculated as the difference between summer and winter temperatures (mean annual range of temperature: MART = WMT−CMT).

## Results

### Temperature Parameters

**Figure 5:**
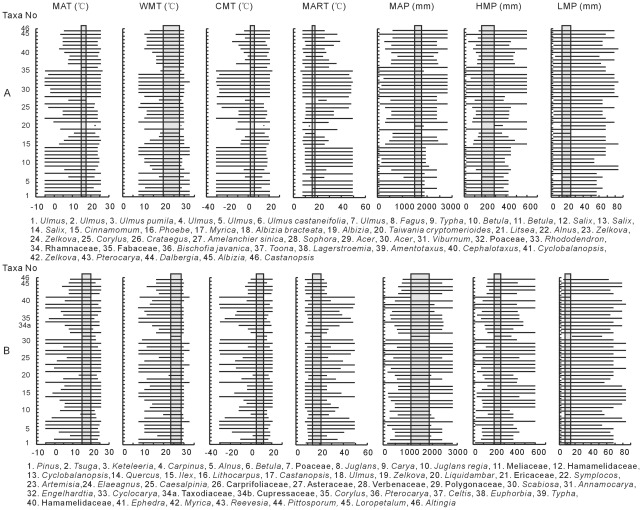
Coexistence intervals of megaflora (A) and palynoflora (B) from Yuanmou Basin. MAT: Mean annual temperature, WMT: Temperature of the warmest month, CMT: Temperature of the coldest month, MART: Mean annual range of temperature, MAP: Mean annual precipitation, HMP: Wettest month precipitation, LMP: Driest month precipitation.

The coexistence intervals of temperature parameters are listed in Table 3 and Fig. 5. The data show that there are some differences in the ranges of mean annual temperature, temperatures of the warmest and coldest months, and mean annual range of temperature obtained from megaflora and palynoflora data. The mean annual temperature estimated from megaflora is 14.8–17.4°C (mean value: 16.1°C)?with the two boundary taxa of *Bischofia javanica* and *Amelanchier sinica*, while the mean annual temperature based on palynoflora (15–19.8°C, mean value: 17.4°C) is?a little bit broader than that of megaflora with the bordering taxa of *Annamocarya* and *Ephedra.* There is an overlapping interval of 15–17.4°C for both.

**Table 3:** Coexistence intervals of megaflora and palynoflora (mean value in the parenthesis).

Climate parameter	Megaflora		Palynoflora	
	Climate value	Bordering taxa	Climate value	Bordering taxa
MAT (°C)	14.8–17.4 (16.1)	*Bischofia javanica–Amelanchier sinica*	15–19.8 (17.4)	*Annamocarya–Ephedra*
WMT (°C)	19.8–27.6 (23.7)	*Bischofia javanica–Albizia bracteata*	23.4–28.6 (26)	*Annamocarya–Scabiosa, Typha,* *Annamocarya, Juglans regia*
CMT (°C)	2–6 (4)	*Bischofia javanica–Amelanchier sinica*	4.9–11.9 (8.4)	*Annamocarya–Ephedra*
MART (°C)	15.6–17.8 (16.7)	*Ulmus pumila–Albizia bracteata*	13.7–21 (17.38)	*Cyclocarya–Annamocarya*
MAP (mm)	1484.3–1784.4 (1634.35)	*Taiwania cryptomerioides–Albizia bracteata*	1114.9–1869.9 (1492.4)	*Annamocarya–Scabiosa*
HMP (mm)	166.4–283.3 (224.85)	*Ulmus castaneifolia*–Fabaceae	198.3–268.1 (233.2)	*Annamocarya–Ephedra*
LMP (mm)	13.2–24.6 (18.9)	*Ulmus castaneifolia–Albizia bracteata*	6.9–14.1 (10.5)	*Carya–Ephedra*

The mean temperatures of the warmest month based on megaflora and palynoflora are 19.8–27.6°C and 23.4–28.6°C, respectively. Although these two ranges are different, they still have an overlapping interval of 23.4–27.6°C. For the mean temperature of the coldest month, two intervals, viz. 2–6°C and 4.9–11.9°C are obtained on the basis of megaflora and palynoflora. The overlapping interval for both is 4.9–6°C. For the mean annual range of temperature, according to the megaflora, a narrow interval of 15.6–17.8°C is obtained, which is determined by lower boundary taxon *Ulmus pumila* and upper one *Albizia bracteata*, while a very wide range of 13.7–21°C is estimated by palynoflora with the lower boundary taxon *Cyclocarya* and upper one *Annamocarya*, encompassing the results from megaflora. Normally, the palynomorphs are identified to the genus and family levels, which permit a correlation with the nearest living relatives at genus and family levels. So pollen data give generally broader coexistence intervals than from the megaflora.

In the megaflora, *Bischofia javanica* plays an important role as a lower boundary taxon for the mean annual temperature (MAT), the mean temperature of the warmest month (WMT), and the mean temperature of the coldest month (CMT). *Amelanchier sinica* acts as the upper boundary taxon for MAT and CMT. *Albizia bracteata* is an upper boundary taxon for WMT and the mean annual range of temperature (MART). In palynoflora, *Annamocarya* is a lower boundary taxon in determining the MAT, WMT and CMT, and it is also an upper boundary taxon for MART. *Ephedra* is considered as the upper boundary taxon for MAT and CMT. In addition, *Scabiosa, Typha* and *Juglans regia* are also very important, being an upper boundary taxon for WMT.

### Precipitation Parameters

The coexistence intervals of precipitation parameters are listed in Table 3 and Fig. 5. The mean annual precipitations based on megaflora and palynoflora are 1484.3–1784.4 mm (mean value: 1634.35 mm) and 1114.9–1869.9 mm (mean value: 1492.4 mm), respectively. *Taiwania cryptomerioides* and *Albizia bracteata* are the two boundary taxa in the megaflora. *Annamocarya* and *Scabiosa* determine the lower and upper borders in palynoflora.

The middle value of wettest month precipitation (HMP) is around 230 mm, ranging from 166.4 mm to 283.3 mm by megaflora and from 198.3 mm to 268.1 mm by palynoflora. For the mean driest month precipitation (LMP), although two intervals are different, viz. 13.2–24.6 mm and 6.9–14.1 mm, there has an overlapping interval of 13.2–14.1 mm for both. In megaflora, *Ulmus castaneifolia* determines the lower borders for HMP and LMP. Fabaceae and *Albizia bracteata* become the upper bordering taxa for HMP and LMP. In palynoflora, *Annamocarya* and *Carya* are the boundary taxa for HMP and LMP. *Ephera* is an upper boundary taxon for both HMP and LMP.

## Discussion

### Comparison of Late Pliocene Climate with other Sites Close to Yuanmou Basin in Yunnan

Previously, some quantitative studies about Late Pliocene climate have been undertaken in Eryuan, Yangyi and Longling of Yunnan Province, Southwest China [26] (Fig. 1A). This enables us to compare them with the data of Yuanmou Basin.

Kou et al. [26] investigated the Eryuan palynoflora from the Late Pliocene of western Yunnan and compared it with two contemporary palynofloras from Yangyi and Longling. Based on this palynological data, the authors quantified the climate of the three localities by using the Coexistence Approach (Table 4). The mean annual temperatures of Longling, Yangyi and Eryuan in the Late Pliocene display a trend from high temperatures to lower ones (mean values: 20.35 to 17.1 to 15.95°C), which fit well with the latitudinal variation, while the mean annual precipitations remain constant from Longling through Yangyi to Eryuan (mean values: 1035.25, 1026.1, and 1052.1 mm, respectively) in the Late Pliocene.

**Table 4:** Comparison of modern and Late Pliocene climates in Yuanmou, Eryuan, Yangyi and Longling, Yunnan Province (The age of Eryuan, Yangyi and Longling are based on a lithostratigraphic and biostratigraphic comparison).

Location	Position and altitude	Time	MAT (°C)	MAP (mm)	References
Yuanmou	25°44′ N, 101°52′ E, 1118.4 m	Modern	21.9	613.8	[38]
Eryuan	26°00′ N, 99°49′ E, 2279 m	Modern	13.9	1078.9	[38], [39]
Yangyi	24°57′ N, 99°15′ E, 1521 m	Modern	15.5	966.4	[38], [39]
Longling	24°41′ N, 98°50′ E, 1802 m	Modern	14.9	2122	[22]
Yuanmou	–	3.4–2.5 Ma	14.8–17.4 (16.1) (megaflora)15–19.8 (17.4) (palynoflora)	1484.3–1784.4 (1634.35)1114.9–1869.9 (1492.4)	Present paper
Eryuan	–	Late Pliocene	13.3–18.6 (15.95)	619.9–1484.3 (1052.1)	[26]
Yangyi	–	Late Pliocene	13.3–20.9 (17.1)	797.5–1254.7 (1026.1)	[26]
Longling	–	Late Pliocene	18.6–22.1 (20.35)	815.8–1254.7 (1035.25)	[26]

Generally, the comparison of our results from Yuanmou with the data of Kou et al. [26] show the climate of central and western Yunnan during the Late Pliocene was warm and humid. The estimated mean annual temperature of Yuanmou is close to those of Eryuan and Yangyi (Yuanmou: 16.1°C (megaflora), 17.4°C (palynoflora), Eryuan: 15.95°C, Yangyi: 17.1°C), which fits well with its geographic position that Yuanmou is located at central Yunnan with a latitude of 25°44′ N between Eryuan (26°00′ N) and Yangyi (24°57′ N) (Fig. 1A). However, mean annual precipitation of Yuanmou is quite different from both of them (Yuanmou: 1634.35 mm (megaflora), 1492.4 mm (palynoflora), Eryuan: 1052.1 mm, Yangyi: 1026.1 mm).

In Yunnan all localities are influenced by the same monsoon system, so it seems to be more likely that the different geometries of the valleys may play a more important role. During the Late Pliocene, in Eryuan, Yangyi and Longling of western Yunnan, the mean annual precipitation (MAP) is around 1000 mm, while in Yuanmou Basin of central Yunnan, the MAP can reach up to ca. 1500–1600 mm. The present values of MAP in Eryuan and Yangyi are about 1000 mm, Longling 2122 mm, Yuanmou 613.8 mm (Table 4). The doubling of the MAP in Longling between the Late Pliocene and the present may be linked to the uplift of Gaoligong Mountain in Longling area which obstructed the moist air-stream northward to Yangyi and Eryuan [26]. The great difference of the MAP in Yuanmou between the Late Pliocene and the present also suggests that some higher mountains raised after the Pliocene and protected the basin from moist air masses.

### The causes for Climatic difference between the Late Pliocene and Today in Yuanmou Basin

The megaflora found in Yuanmou Basin includes a large number of tropical and subtropical plants, viz., *Albizia*, *Bischofia*, *Castanopsis*, *Cinnamomum*, *Cyclobalanopsis*, *Litsea*, *Phoebe* and *Taiwania*, and some temperate plants, viz., *Acer, Alnus*, *Betula*, *Salix*, *Ulmus* and *Zelkova.* Similarly, the palynoflora also comprises abundant tropical and subtropical plants, viz., *Altinigia*, *Caesalpinia*, *Castanopsis*, *Lithocarpus*, Meliaceae, *Pittosporum* and *Symplocos*, temperate and subtropical plants, viz., *Annamocarya, Carya* and *Liquidambar*, and temperate plants, viz., *Alnus*, *Betula*, *Celtis*, *Juglans*, *Pinus*, *Polygonum*, *Ulmus* and *Zelkova* (Tables 1, 2). Both of the megaflora and palynoflora suggest a warm and humid subtropical climate condition [7], [11], [12].

The modern climate in Yuanmou Basin is of a southern subtropical type. For the Late Pliocene, the quantitative data of temperature and precipitation suggest that the climate was warm and humid and demonstrate generally subtropical conditions in Yuanmou Basin also at that time. The mean annual temperature of Late Pliocene is about 5°C lower than the present, while the mean annual precipitation at that time is about 2.5 times of today. The possible reasons behind the difference of Late Pliocene and modern climates in Yuanmou Basin may be explained as follows. From the view of global climatic change, the Pliocene represents a transition from a relatively warm climate stage into the icehouse of the Pleistocene due to the growth of large terrestrial ice sheets and the onset of Northern Hemisphere glaciation [27], which is believed to be partially affected by long-term periodic variations in incoming solar radiation [28]. The Pliocene-Pleistocene global climate displays a cooling trend [29]–[31], while the present study shows a warming from the Late Pliocene (ca. 16–17°C) to the present (21.9°C) in Yuanmou Basin. So the climate difference seems not due to global climatic change. Then the local tectonic change and monsoon activity should be considered. The Yuanmou Basin was initiated at ca 3.5 Ma as a syncline basin and completed as an asymmetric half-graben after ca 1.1Ma with the movement of the Yuanmou-Dongshan Fault, an eastern marginal fault of the basin. The relative subsidence of the basin ended during the early Middle Pleistocene (less than 780 ka), in concordance with the tectonic event around the Tibetan Plateau [32]. Thus, the closed dry-hot valley of Yuanmou was formed with a higher altitude in the eastern side and a lower altitude in the western side. As far as the monsoon activity is concerned, the Indian summer monsoon displayed a general trend of gradual intensification during the Late Pliocene (3.57–2.78 Ma) based on a high-resolution terrestrial grain-size record from the Yuanmou Basin [21]. Moreover, the East Asian summer monsoon also strengthened at ca. 3.5–2.5 Ma supported by the sediment record in the South China Sea [33] as well as by other independent palaeoclimatic evidences [34]–[37]. Based on both considerations, it seems to be obvious that no barrier like high mountains existed in the central part of Yunnan and monsoon strengthened during the Late Pliocene. So the moist air masses from the Indian Ocean could have penetrated into the Yuanmou Basin and brought abundant rainfall. However, now it is a closed dry-hot valley (Fig. 4) and the foehn effect contributes to a temperature and evaporation rise. Thus, the local climatic situation during that time was quite different from the dry, hot climate conditions of today.

In the future, we will attempt to reconstruct the changes in climate and environment in the transition from Late Pliocene to and during Early Pleistocene in the Yuanmou Basin for a better understanding of the environmental context of Yuanmou Man.
